# New records for chewing lice of the genus *Dennyus* Neumann, 1906 (Phthiraptera: Amblycera) on two swifts from Saudi Arabia

**DOI:** 10.3897/BDJ.9.e67927

**Published:** 2021-08-05

**Authors:** Kholoud A. Al-Shammery

**Affiliations:** 1 Department of Biology, College of Science, Ha’il University, 1441 Ha’il, Saudi Arabia Department of Biology, College of Science, Ha’il University 1441 Ha’il Saudi Arabia

**Keywords:** ectoparasites, wild birds, chewing lice, new record

## Abstract

**Background:**

Very little is known about the chewing lice fauna of Saudi Arabia especially from swifts (Apodidae). Swifts are common resident and migratory birds throughout Saudi Arabia. Two chewing lice genera are known for parasitising swifts throughout the world: *Dennyus* Neumann, 1906 and *Eureum* Nitzsch, 1818, none of which have been recorded from the Kingdom before.

**New information:**

Two species of resident wild swifts were examined for chewing lice for the first time in Saudi Arabia. Two rare lice species were identified: D. (Dennyus) hirundinis (Linnaeus, 1761) and *Dennyus* sp. (Phthiraptera: Amblycera: Menoponidae) infesting the common swift *Apusapus* (Linnaeus, 1758) and African palm swift *Cypsiurusparvus* (Lichtenstein, 1823), respectively. The described chewing lice species are considered as new country records. They will be added to the Saudi Arabia parasitic fauna. Taxonomical and ecological remarks were provided for the identified chewing lice through this work, along with notes on swift/chewing lice interaction.

## Introduction

Swifts (Apodiformes: Apodidae) are one of the fastest bird groups in the world and includes around 113 species (Gill et al. 2021). Some species can fly with a speed of about 111.96 km/h (Henningsson et al. 2010). They are insectivorous birds that catch small insects during their fast-flying (Chantler 2010). Their flying mechanism and piercing vision make catching and examining this group of birds challenging. In Saudi Arabia, there are six recorded species of swifts, all of Palearctic and Afrotropical origin (Gill et al. 2021).

The multiple origins of birds of Saudi Arabia reflects the diversified avifauna of the Kingdom, but with such great diversity, the birds’ ectoparasites of the country are far from understood, especially the chewing lice (Nasser et al. 2020). The number of chewing lice species, recorded in the last decade in Saudi Arabia, is not representative of the great avifauna of the country (El-Ahmed et al. 2012, Al-Ahmed et al. 2014, Alahmed et al. 2015, Shobrak et al. 2015, Nasser et al. 2015, Nasser et al. 2015, Nasser et al. 2016, Alahmed et al. 2017). In Saudi Arabia, swifts have never been examined for chewing lice, but worldwide, there are two chewing lice genera that are known to infest swifts: *Dennyus* and *Eureum* (Price et al. 2003). Several lice species are expected to be associated with Saudi Arabia’s swifts (Table 1).

Chewing lice species of the genus *Dennyus* occur on swifts with low prevalence (Price and Clayton 1997). Harrison 1916 tried to clarify the status of this genus and he reported six valid species of it across the world, but the genus today includes 49 species belonging to four subgenera: *Collodennyus* Ledger, 1970, *Ctenodennyus* Ewing, 1930, *Dennyus* sensu stricto Neumann, 1906 and *Takamatsuia* Uchida, 1926 (Clayton et al. 2006, Valim 2013). From a taxonomic point of view, *Dennyus* is characterised by a characteristic head shape that lacks oesophageal sclerite, prothorax very narrow and the hind femora with a patch of spine on the ventral face, also the 6^th^ and 8^th^ abdominal sternites with spiny patches; it resembles the genus *Myrsidea* even in male genitalia (Ferris 1916). The genus *Dennyus* has never been recorded before in the Middle East. Therefore, exploration of such neglected ectoparasites of a special bird group, such as swifts, is essential to describe parasite diversity in the region (Adly et al. 2019, Adly et al. 2020).

Through the present work, the aim is to update the knowledge about the chewing lice infesting the swifts of Saudi Arabia and add two new records of chewing lice to the Kingdom's parasitic fauna.

## Materials and methods

**Figure 1.**
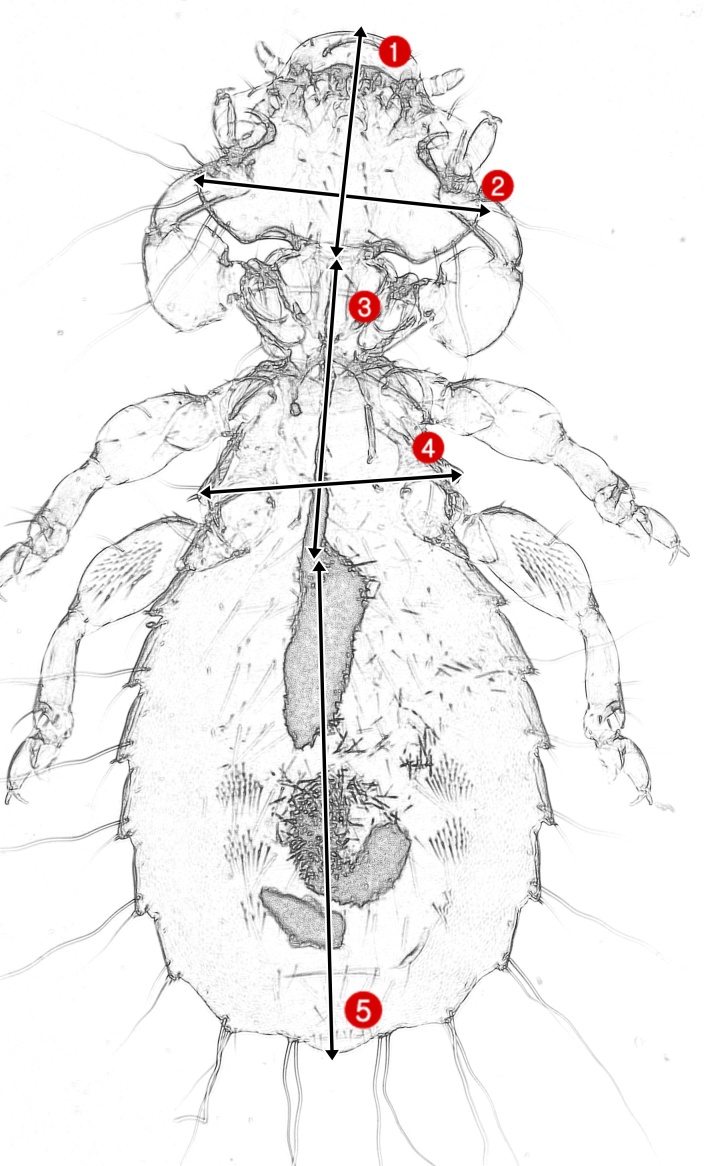
Illustrative model of genus *Dennyus* showing the way by which each measurement were taken; 1. Head Length = HL; 2. Head Width = HW; 3. Thorax Length = TL; 4. Thorax Width = TW and 5. Abdomen Length = AL.

Two species of swifts: the African palm swift, *Cypsiurusparvus* and the common swift, *Apusapus* were examined for chewing lice. One African palm swift was examined in Sabia, Jazan (17°8'1"N; 42°37'17"E) and three common swifts were examined at Aja Mountain, Ha’il (27°28'58"N; 41°36'50"E); however, only the African palm swift and one common swift were infested with lice. A standard mist net: (mesh: 1.8 cm × 0.12 mm and size: 2 m × 15 m) was used for capturing the examined birds. The live caught birds were first inspected visually, then a fumigation chamber was used for chewing lice collection using chloroform as a fumigant for 3 min (Clayton and Drown 2001). Following fumigation, lice were removed by ruffling over on to a white surface. The captured birds were immediately released safely at the place of capture. The collected chewing lice were preserved in 95% ethyl alcohol, then cleared using lactic acid for 2 days. Finally, specimens were mounted by using Puri’s media (Nasser et al. 2019). Chewing lice species identification was done according to Kellogg and Paine (1914), Ewing (1930), Thompson (1948). A S-EYE YW500 camera5mp was used in specimens’ photographs. Photoshop Lightroom 5 (Adobe Systems Inc., San Jose, CA) was used to prepare final images (Nasser et al. 2015). All specimens are kept in Ha'il University Zoological Collection. Samples were measured using microscopic software according to the provided illustration (Fig. 1). Measurements are abbreviated as follows: Head Length = HL; Head Width = HW; Head Index = HI; Thorax Length = TL; Abdomen Length = AL; Total Length = TOL. Ecological and taxonomic remarks on recorded lice species are included.

## Taxon treatments

### Dennyus sp.

8987A3D5-2EB4-573C-AB78-D672D958DE7A

#### Materials

- **Type status:**Other material. **Occurrence:** recordedBy: Kholoud A. Al-Shammery; individualCount: 1; lifeStage: 1 nymph; **Taxon:** kingdom: Animalia; phylum: Arthropoda; class: Insecta; order: Phthiraptera; family: Menoponidae; genus: Dennyus; specificEpithet: sp.; **Location:** country: Saudi Arabia; stateProvince: Sabia, Jazan; **Event:** eventDate: February/2018

#### Description

**Figure 2.**
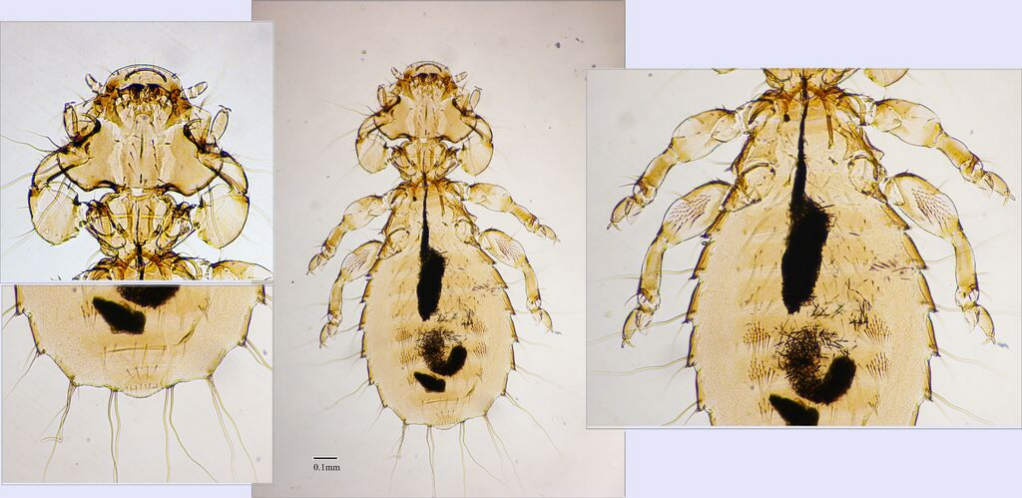
Nymph of *Dennyus* sp.; upper left: Head; lower left: Genital region; right side: close up to the body; the scale bar is only applicable to the full body image.

Head roughly triangular, third wider than long, antenna along with a large fourth segment, temple rectangular without any chitinisation, gula weakly developed, with a row of relatively long seta; prothorax small broader than long, meso and meta thorax equal in length with acute lateral margin, meta thorax with two small spine-like seta on each lateral margin; forelegs with flattened enlarged femora and large coxa, as characteristic of the genus, hind femora with patch medium-sized brush; abdomen rectangular without pigmentation, first segment with transverse characteristic chitinisation, lateral margin rounded with five very small thorns like seta on segment III-V; segment IV and V with a clear ventral brush; male terminalia much narrower (Fig. 2).

**Measurements**: Female HL: 0.39; HW: 0.53; HI: 0.74; TL: 0.53; AL: 0.95; TOL: 1.87 ± 0.2.

#### Distribution

Afrotropical

#### Ecology

**Local host**: African palm swift *Cypsiurusparvus* (Lichtenstein, 1823).

**Known hosts**: *Cypsiurusparvus* (Lichtenstein, 1823), African palm swift.

#### Notes

From my examination and description, as well as the host/parasite association, this species could be D. (Dennyus) cypsiurus and, in this case, it will be the first record from Saudi Arabia. Not much is known about that species. The collected samples were found on the body feathers near the anus of the host.

### Dennyus (Dennyus) hirundinis

Linnaeus, 1761

B23B1ED6-A010-50A4-A509-5CF9C0A909AE

Dennyus (Dennyus) hirundinis **Dennyus (Dennyus) hirundinis(L., 1761:479)**
Pediculus hirundinis Linnaeus, 1761:479
Nirmus truncatus Olfers, 1816:91
Nitzschia burmeisteri Denny, 1842:202
Nitzschia tibialis Piaget, 1880:576
Dennyus africanus Büttiker, 1954:159
Dennyus clayae Nakagawa, 1959a:164
Dennyus maritimus Buttiker, 1954:160
Nitzschia minor Kellogg & Pame, 1914:242
Dennyus minutus Buttiker, 1954:160
Dennyus truncatiformis Mokhehle, 1951:341

#### Materials

- **Type status:**Other material. **Occurrence:** recordedBy: Kholoud A. Al-Shammery; individualCount: 1; sex: 1 female; lifeStage: 1 adult; **Taxon:** kingdom: Animalia; phylum: Arthropoda; class: Insecta; order: Phthiraptera; family: Menoponidae; genus: Dennyus; subgenus: Dennyus; specificEpithet: *hirundinis*; **Location:** country: Saudi Arabia; stateProvince: Aja Mountain, Ha’il; **Event:** eventDate: February 2018

#### Description

**Figure 3.**
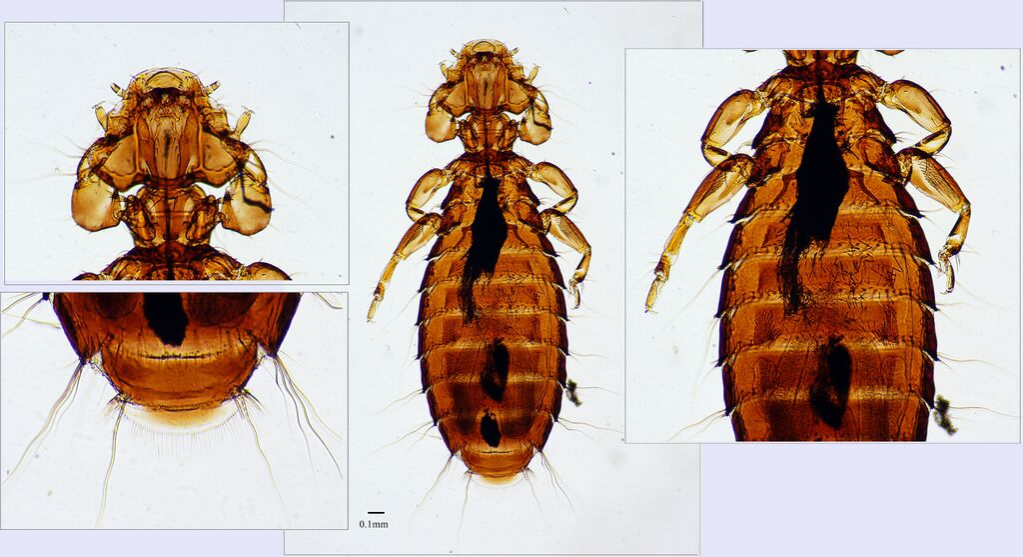
Female of Dennyus (Dennyus) hirundinis Linnaeus; upper left: Head; lower left: Genital region; right side: close up to the body; the scale bar is only applicable to the full body image.

Head trilobed triangular in shape, maxillary palp protruding out of the head, antenna very short, temple rectangular with characteristic highly chitinized out line, gula well developed with lateral row of four black spin-like seta and characteristic chitinization surrounding; thorax clearly divided, prothorax small quadrat shape, meso and meta thorax highly chitinized on lateral margin with acute ends; fore legs with large coxa and enlarged rounded femora, mid and hind legs slightly equal in length, ventral side of hind femora covered with elongated brush of short seta; abdomen elongated oval in shape with highly chitinized lateral margin with acute ends and two small thorn-like seta, the ventral brush appears in segment IV and V; the body of this species is highly pigmented (Fig. 3).

**Measurements**: Female HL: 0.47; HW: 0.64; HI: 0.73; TL: 0.57; AL: 1.94; TOL: 2.98 ± 0.2.

#### Distribution

Dennyus (Dennyus) hirundinis distribution: Palaearctic: Asia (Afghanistan, China, India, Malaysia, Sri Lanka, Thailand); Europe (England, Germany, Scotland, Spain, Switzerland, Turkey); Afrotropical: Africa (Cameroons, Cape Verde, Congo, Kenya, Nigeria, Somalia, South Africa, Zimbabwe).

#### Ecology

**Local host**: only one sample was collected from common swift, *Apusapus*.

**Known hosts**: *Apusapus* (L., 1758), common swift (type host); *Aerodramusunicolor* (Jerdon, 1840), Indian swiftlet; *Apusalexandri* Hartert, 1901, Alexander´s swift; *Apuspallidus* (Shelley, 1870), Pallid swift; *Apusbarbatus* (Sclater, 1866), African swift; *Apusbradfieldi* (Roberts, 1926), Bradfield´s swift; *Apuspacificus* (Latham, 1801), fork-tailed swift; *Apusacuticauda* (Jerdon, 1864), dark-rumped swift; *Apusaffinis* (Gray, 1830), little swift; *Apushorus* (Heuglin, 1869), Horus swift; *Apuscaffer* (Lichtenstein, 1823), white-rumped swift.

#### Notes

This report constitutes a new geographical record of D. (Dennyus) hirundinis from Saudi Arabia. This species is found throughout the body of the host; the collected sample was found feeding on the eye fluid of the host during the night.

## Discussion

Recently, several publications have focused on the taxonomic status of chewing lice infesting domestic, resident, migratory and exotic birds of Saudi Arabia (El-Ahmed et al. 2012, Al-Ahmed et al. 2014, Alahmed et al. 2015, Shobrak et al. 2015, Nasser et al. 2015, Nasser et al. 2015, Nasser et al. 2016, Alahmed et al. 2017). Although these works greatly improve our knowlege of the chewing lice fauna of Saudi Arabia, they represent very small steps in a long path to achieving a comprehensive understanding of the real status of such an interesting insect group throughout the country. Many bird groups have never been examined for chewing lice and a huge number of lice species wait to be recorded in Saudi Arabia for the first time or described even as a new species (Nasser et al. 2016). One such neglected group is swifts; this paper represents the first study of swift lice in Saudi Arabia. Although the swifts are very common birds in many parts of the Kingdom, catching them alive is a very difficult task that limits our ability to study their ectoparasites, including chewing lice.

There are two genera of chewing lice that are known to infest swifts, neither of which had been previously recorded from Saudi Arabia before. The current study represents the first published records of *Dennyus* species from Saudi Arabia and the region. Therefore, the record of D. (Dennyus) hirundinis and unidentified *Dennyus* nymph are considered an addition to Saudi Arabia's parasitic fauna. Although *Dennyus* spp. are generally characterised by high degrees of host specificity (Tompkins and Clayton 1999), some of its species are recorded from several swift species (Price et al. 2003, Clayton et al. 2006); such facts prevent the identification of an unidentified species of chewing lice here as D. (Dennyus) cypsiurus Thompson, 1948 which is already known to infest African palm swift. There are likely more species of *Dennyus* waiting to be recorded from Saudi Arabia as additional louse samples from swifts become available (Gill et al. 2021).

The two species of swifts that have been examined in this work (the African palm swift, *C.parvus* and the common swift, *A.apus*) are considered migratory birds (Jennings 2010). This fact raises a lot of questions about the impact of bird migration on chewing lice and if migratory birds are transferring lice species to resident and domestic birds. To answer such questions we must, first, obtain a clearer understanding of the chewing lice fauna of both resident and migratory species within Saudi Arabia. The new records reported here help with this task.

## Supplementary Material

XML Treatment for Dennyus sp.

XML Treatment for Dennyus (Dennyus) hirundinis

## Figures and Tables

**Table 1.** Summary of swift species (Apodiformes) encountered in Saudi Arabia with their expected associated chewing lice, according to Price et al. (2003).

**Swift species**	**Louse species**
Alpine swift, *Tachymarptismelba* (L., 1758)	Dennyus (Dennyus) vonarxi Büttiker, 1954
Common swift, *Apusapus* (L., 1758)	Dennyus (Dennyus) hirundinis (L., 1761) *Eureumcimicoides* Burmeister, 1838
Little swift, *Apusaffinis* (JE Gray, 1830)	Dennyus (Dennyus) hirundinis (L., 1761) *Eureumcimicoides* Burm., 1838
Pallid swift, *Apuspallidus* Shelley, 1870	Dennyus (Dennyus) hirundinis (L., 1761)
White-rumped swift, *Apuscaffer* (Lichtenstein, 1823)	Dennyus (Dennyus) hirundinis (L., 1761) *Eureumpygostyli* Mokhehle, 1951
African Palm-Swift, *Cypsiurusparvus* (Lichtenstein, 1823)	Dennyus (Dennyus) cypsiurus Thompson, 1948
